# Minimally invasive *versus* open synchronous colorectal and hepatic resection for metastatic colorectal cancer: American College of Surgeons National Surgical Quality Improvement Program (ACS NSQIP) analysis

**DOI:** 10.1093/bjsopen/zrad149

**Published:** 2023-12-07

**Authors:** Matthew C Lund, Laura J Allen, Juan G Glinka, Elizabeth M Shin, Douglas Quan, Anton I Skaro, Ephraim S Tang

**Affiliations:** Schulich School of Medicine and Dentistry, Western University, London, Ontario, Canada; London Health Sciences Centre, University Hospital, London, Ontario, Canada; Schulich School of Medicine and Dentistry, Western University, London, Ontario, Canada; London Health Sciences Centre, University Hospital, London, Ontario, Canada; Schulich School of Medicine and Dentistry, Western University, London, Ontario, Canada; London Health Sciences Centre, University Hospital, London, Ontario, Canada; Schulich School of Medicine and Dentistry, Western University, London, Ontario, Canada; London Health Sciences Centre, University Hospital, London, Ontario, Canada; Schulich School of Medicine and Dentistry, Western University, London, Ontario, Canada; London Health Sciences Centre, University Hospital, London, Ontario, Canada; Schulich School of Medicine and Dentistry, Western University, London, Ontario, Canada; London Health Sciences Centre, University Hospital, London, Ontario, Canada; Schulich School of Medicine and Dentistry, Western University, London, Ontario, Canada; London Health Sciences Centre, University Hospital, London, Ontario, Canada

## Introduction

Colorectal cancer (CRC) is the fourth leading cause of deaths from cancer worldwide and is the third most common cancer in North America^1–3^. About 30% of patients with CRC will have metastasis to the liver and 15% of patients have synchronous liver metastases at the time of initial diagnosis^4,5^. Resection of liver metastases is the best method for improving long-term survival in patients with metastatic disease^6–8^. Traditionally, this was done sequentially, with resection of the primary tumour performed separately to the resection of liver metastases^8,9^. However, synchronous resection has now become popular, as improved surgical technique and close cooperation between colorectal and hepatopancreatobiliary surgeons have resulted in an increase in the safety of this approach^8,10–12^. Although data are limited, the synchronous approach has been found to be safe^8,11^. Beyond the clear advantages of a single surgery, wound morbidity is often higher with combined resections, as they require a full-length laparotomy incision for access to both the pelvis and upper abdomen. An attractive strategy that avoids this added morbidity is the application of minimally invasive surgery (MIS) and, although this approach has been reported, case counts remain low and data on safety are lacking^13–17^.

The American College of Surgeons (ACS) National Surgical Quality Improvement Program (NSQIP) gathers de-identified patient data from participating hospitals and contains more than 6.6 million cases from approximately 700 sites across North America. Previous work using this database has shown that synchronous resection is associated with increased major morbidity compared with staged resection^18^. However, no differentiation was made between open and minimally invasive synchronous resection. The aim of this study was to perform a propensity score matched analysis, leveraging the power of the large NSQIP procedure targeted database, to examine the difference between minimally invasive and open combined colorectal and hepatic resection.

## Methods

A retrospective cohort study comparing minimally invasive and open combined colorectal and liver resections was completed using prospectively collected data from the ACS NSQIP database. Participant user files (PUFs) and hepatectomy procedure targeted PUFs were obtained for the years 2014–2021. Data sets were combined using common patient IDs. Patients with current procedural terminology (CPT) codes for both hepatic and colorectal resections were identified. Patients undergoing emergency surgery were excluded, as were ASA grade V patients and patients who were dependent on a ventilator before surgery. Hepatectomies were classified as minor or major based on their CPT codes. Similarly, colorectal resections were categorized as right colectomy, left colectomy, or proctectomy. Please see the *Supplementary material* for a detailed explanation and full breakdown of the CPT codes used for categorization.

Demographics and patient outcomes were compared using independent *t* tests, Mann–Whitney *U* tests, and chi-squared tests, as appropriate. The primary outcome was combined 30-day major morbidity, defined as the presence of one or more of the following complications: stroke, cardiac arrest, myocardial infarction, deep-vein thrombosis, pulmonary embolism, sepsis, prolonged ventilation, deep surgical site infection (SSI), organ space SSI, wound disruption, unplanned intubation, and unplanned reoperation. Secondary outcomes included 30-day mortality rate, duration of operation, duration of hospital stay, and rates of postoperative bile leak and liver failure.

Propensity score matching was performed using complete cases based on a logistic regression model fitted using clinically important covariates (please see the *Supplementary material*). Each MIS patient was matched with two open surgery patients using nearest neighbour matching without replacement. Calipers were set to 0.2 times the standard deviation of the logit of the propensity score^19^. The quality of matching was assessed using standardized mean differences presented as a love plot, as well as mirrored histograms of the logit of the propensity score.

Statistical significance was set at *P* < 0.050 for all analyses. Propensity score matching was completed using the R MatchIt package. All other analysis was completed using SPSS^®^ (IBM, Armonk, NY, USA; version 26.0, released 2019).

Finally, logistic regression was completed on the unmatched cohort to determine the independent odds of major morbidity in patients undergoing open *versus* minimally invasive resections, controlling for age, sex, ASA grade, type of colorectal resection, and extent of liver resection.

## Results

**Fig. 1:**
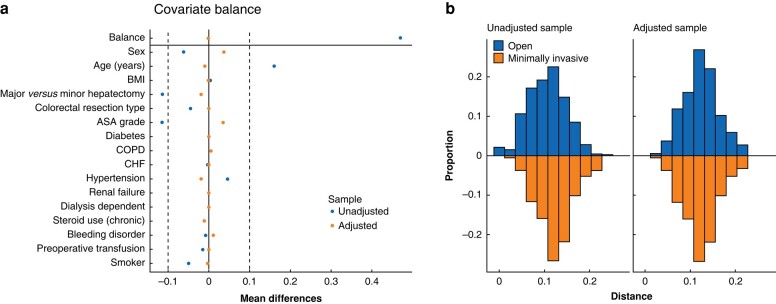
Propensity score matching **a** Love plot illustrating covariate balance before and after propensity score matching. After matching, there was reduced variability among many confounding factors between the minimally invasive and open populations. COPD, chronic obstructive pulmonary disease; CHF, congestive heart failure. **b** Mirrored histograms confirming improved distributional balance for propensity scores after matching.

Between 2014 and 2021, a total of 1561 patients undergoing totally open surgery and 187 patients undergoing totally MIS were identified. After 2 : 1 propensity score matching, 369 patients undergoing open surgery and 186 patients undergoing MIS were included. Demographic data and surgical characteristics are provided in *Table 1*. Before matching, patients in the open surgery group tended to have more co-morbidities and were more likely to have major hepatectomies. After matching, there was excellent overlap between the groups (*Fig. 1*).

**Table 1:** Patient demographics and surgical characteristics before and after matching

Variable	Before matching			After matching		
	MIS (*n* = 187)	Open surgery (*n* = 1561)	*P*	MIS (*n* = 186)	Open surgery (*n* = 369)	*P*
Age (years), mean(s.d.)	61.1(12.1)	59.2(12.4)	0.052	61.0(12.1)	61.0(11.8)	0.989
**Sex**						
Male	106	793	0.141	105	221	0.437
Female	81	768		81	148	
BMI (kg/m^2^), mean(s.d.)	27.9(6.4)	27.9(5.9)	0.944	27.9(6.4)	28.0(5.6)	0.699
**ASA grade**						
I	1	6	0.452	1	1	0.929
II	45	319		44	94	
III	131	1108		131	255	
IV	10	124		10	19	
**Diabetes**						
No	158	1320	0.98	157	311	0.969
Yes	29	241		29	58	
**Smoking status**						
No	166	1308	0.077	165	326	0.899
Yes	21	253		21	43	
**Dyspnoea**						
No	152	1337	0.161	151	307	0.674
Yes	13	74		13	19	
Unknown				22	43	
**COPD**						
No	181	1521	0.602	180	359	0.732
Yes	6	40		6	10	
**Ascites**						
No	186	1540	0.347	185	366	0.717
Yes	1	21		1	3	
**CHF**						
No	187	1554	0.396	186	369	NA
Yes	0	6		0	0	
**Hypertension**						
No	104	937	0.246	104	200	0.702
Yes	83	624		82	169	
**Acute renal failure**						
No	187	1559	0.624	186	369	NA
Yes	0	2		0	0	
**Preoperative steroids**						
No	182	1502	0.447	181	355	0.499
Yes	5	59		5	14	
**Extent of liver resection**						
Major	25	385	<0.001	25	57	0.53
Minor	162	1176		161	312	
**Type of colorectal resection**						
Right	65	466	0.226	64	115	0.355
Left	60	594		60	142	
Proctectomy	62	501		62	112	
**Combined resection type**						
Minor hepatectomy						
Right colectomy	57	341	0.225	56	93	0.292
Left colectomy	51	435		51	121	
Proctectomy	54	387		54	98	
Major hepatectomy						
Right colectomy	8	121	0.886	8	22	0.753
Left colectomy	9	154		9	21	
Proctectomy	8	107		8	14	

Values are *n* unless otherwise indicated. MIS, minimally invasive surgery; COPD, chronic obstructive pulmonary disease; CHF, congestive heart failure; NA, not applicable.

Major morbidity occurred in 20 patients in the MIS group (10.8%), compared with 91 patients (24.7%) in the open surgery group (*P* < 0.001) (See *Table S1* for breakdown of major morbidity). The mortality rate was low in each group (1.1% in the MIS group (2 of 186 patients) *versus* 1.6% in the open surgery group (6 of 369 patients); *P* = 0.602). The median duration of hospital stay was shorter in the MIS group compared with the open surgery group (5 *versus* 7 days respectively; *P* < 0.001), whereas the median duration of operation was similar between the groups (318 min in the MIS group *versus* 305 min in the open surgery group; *P* = 0.084). There was no difference in postoperative bile leak or liver failure between the groups. Bile leak occurred in 5 patients (2.7%) in the MIS group and in 4 patients (1.1%) in the open surgery group (*P* = 0.189), whereas liver failure occurred in 6 patients (3.2%) in the MIS group and in 20 patients (5.4%) in the open surgery group (*P* = 0.248). On logistic regression, the open surgery approach was independently associated with a significantly increased rate of major postoperative morbidity (OR 2.70, 95% c.i. 1.67 to 4.38; *P* < 0.001) (*Table S2*).

## Discussion

The present study used the large NSQIP database to show that the MIS approach to synchronous resection of CRC with liver metastasis is associated with a significant reduction in postoperative major morbidity compared with the open surgery approach. Despite the technical difficulty of MIS hepatectomies, there was no increase in postoperative bile leak or liver failure, as well as no difference in median duration of operation between the two groups.

A major challenge in retrospective analyses comparing MIS and open surgery is inherent selection bias, with a tendency to perform open surgery for more challenging cases and to favour MIS approaches in patients with less extensive disease requiring smaller resections. The present study addressed this in two ways. First, the present study used logistic regression to show that the MIS approach is independently associated with reduced morbidity, while controlling for potential confounders (including extent of resection and ASA grade). Second, the present study sought to minimize demographic variability by performing a propensity score matched analysis. After adjusting for both demographic factors and extent of resection, a reduction in postoperative major morbidity was still strongly supported. These results support earlier single-centre studies demonstrating reduced major postoperative morbidity with minimally invasive resection compared with the open surgery approach^10,15^.

The present study is limited by its retrospective nature and by the small number of patients undergoing MIS. Although this is a low-volume operation, a greater number of such patients were expected to be identified; some were likely missed due to their inclusion in colectomy procedure targeted PUFs rather than hepatectomy procedure targeted PUFs. No data could be incorporated from the colectomy procedure targeted PUFs, as they contain no information on whether the concurrent hepatic resections were performed using an open surgery approach or an MIS approach. It is unclear how many cases could not be accessed because of this.

## Supplementary Material

zrad149_Supplementary_Data

## Data Availability

Data used in this study are available via the American College of Surgeons National Surgical Quality Improvement Program (ACS NSQIP).
