# Supplementary material for: Minimally invasive versus open synchronous colorectal and hepatic resection for metastatic colorectal cancer: American College of Surgeons National Surgical Quality Improvement Program (ACS NSQIP) analysis
Source: BJS Open. 2023 Dec 7;7(6):zrad149. doi: 10.1093/bjsopen/zrad149 (PMC10702620; doi:10.1093/bjsopen/zrad149)
Supplement: zrad149_Supplementary_Data [file zrad149_supplementary_data.docx]

**Minimally Invasive vs Open Synchronous Colorectal and Hepatic Resection for Metastatic Colorectal Cancer: An ACS NSQIP Analysis**

Matthew C Lund, MD.^1,2^, Laura J Allen, MSc. ^1,2^, Juan G Glinka, MD. ^1,2^, Elizabeth M Shin, MD. ^1,2^, Douglas Quan, MD. ^1,2^, Anton I Skaro, MD. ^1,2^, Ephraim S Tang, MD. ^1,2^

^1^ Schulich School of Medicine and Dentistry, Western University, London ON Canada

^2^ London Health Sciences Centre

Dr. Ephraim S. Tang, MD

Assistant Professor, Department of General Surgery

Schulich School of Medicine and Dentistry

[ephraim.tang@lhsc.on.ca](mailto:ephraim.tang@lhsc.on.ca)

London Health Sciences Centre – University Hospital

339 Windermere Road

London Ontario N6A 5A5

Office Phone: 519.663.3290

Office Fax: 519.663.3858a

**Supplementary Materials - Index**

| **Supplementary Methods** |  |
| --- | --- |
| Detailed methods | *pag. 2* |
| **Supplementary Figures and Tables** |  |
| Supplemental Table 1  Supplemental Table 2 | *pag. 3*  *pag. 4* |
|  |  |
|  |  |

**Supplementary Methods**

Minimally invasive hepatectomies were identified using the “HEP_APPROACH” variable available in the Hepatectomy Procedure Targeted PUF. Robotic cases were included in the minimally invasive group. Hepatectomies were classified as minor or major based on their CPT codes. Trisegmentectomy (47122), Total left lobectomy (47125), and Total right lobectomy (47130) were classified as major hepatectomies^8^. Partial lobectomy (47120) was classified as a minor hepatectomy.A similar process was used to categorize colorectal resections into Right colectomy (44160, 44205) Left colectomy (441401 44141, 44143, 44144, 44204, 44206), or Proctectomy (44145, 44146, 44147, 45111, 45112, 45114, 44119, 44150, 44151, 44155, 44156, 44157, 44158, 45110, 44207, 44208, 44210, 44211, 44212). Cases which had multiple concurrent colorectal or hepatic resections coded within the dataset were categorized based on the most extensive resection performed. Some open cases included both minimally invasive and open colectomy CPT codes. These cases were excluded from the final analysis as there is no mechanism by which it is possible to determine whether these cases represented conversion to open or whether they were simply miscoded. Cases which were specifically coded as laparoscopic with unplanned conversion to open were included in the laparoscopic group.

Liver and colorectal resections for CRC vary widely based on location of the primary tumour and size and number of liver metastases. In addition, advanced liver and colorectal lesions are less amenable to a minimally invasive approach and there is probably substantial selection bias in the cases selected for minimally invasive or open approaches even in expert centers. We used propensity score matching to correct for the physiologic impact of these procedures and reduce selection bias. Propensity score matching was performed using complete cases based on a logistic regression model fitted using clinically important covariates. Covariates included in the model were age, sex, BMI, extent of liver resection (major vs minor), type of colorectal resection, ASA class, and the following comorbidities: diabetes, chronic obstructive pulmonary disease (COPD), congestive heart failure (CHF), hypertension, renal failure, dialysis dependence, chronic steroid use, bleeding disorders, pre-operative transfusion requirement, and smoking. Each minimally invasive case was matched with two open cases using nearest neighbour matching without replacement. Calipers were set to 0.2 times the standard deviation of the logit of the propensity score. The quality of matching was assessed using standardized mean differences presented as a Love Plot (Figure 1A) as well as mirrored histograms of the logit of the propensity score (Figure 1B). Outcomes of interest were re-examined using Mann-Whitney-U tests and chi-squared tests, as appropriate.

**Supplementary Figures and Tables**

| **Table S1: Major Morbidity Breakdown**   \| Variable \|  \| Laparoscopic \| Open \| *p* \| \| --- \| --- \| --- \| --- \| --- \| \|  \| N = 186 \| N = 369 \| \| Complications *n* (%) \| \|  \|  \|  \| \|  \| Stroke \| 0 \| 1 \| 0.477 \| \|  \| Cardiac Arrest \| 0 \| 1 \| 0.477 \| \|  \| MI \| 1 \| 5 \| 0.379 \| \|  \| DVT \| 4 \| 10 \| 0.692 \| \|  \| Sepsis \| 5 \| 28 \| 0.021 \| \|  \| Prolonged Ventilation \| 3 \| 11 \| 0.332 \| \|  \| Deep SSI \| 1 \| 3 \| 0.717 \| \|  \| Organ Space SSI \| 13 \| 57 \| 0.005 \| \|  \| Wound Disruption \| 0 \| 5 \| 0.111 \| \|  \| Unplanned Intubation \| 3 \| 12 \| 0.261 \| \|  \| PE \| 1 \| 13 \| 0.034 \| \|  \| Reoperation \| 2 \| 22 \| 0.008 \| |  |  |  |
| --- | --- | --- | --- | --- | --- | --- | --- | --- | --- | --- | --- | --- | --- | --- | --- | --- | --- | --- | --- | --- | --- | --- | --- | --- | --- | --- | --- | --- | --- | --- | --- | --- | --- | --- | --- | --- | --- | --- | --- | --- | --- | --- | --- | --- | --- | --- | --- | --- | --- | --- | --- | --- | --- | --- | --- | --- | --- | --- | --- | --- | --- | --- | --- | --- | --- | --- | --- | --- | --- | --- | --- | --- | --- | --- | --- | --- |


**Table S2: Logistic regression model of independent predictors of major morbidity. Major hepatectomy and an open surgical approach are independently associated with increased major morbidity.**

|  |  |  |  | 95% C.I. | |
| --- | --- | --- | --- | --- | --- |
|  |  | Sig | OR | Lower | Upper |
| Age |  | 0.008 | 1.013 | 1.003 | 1.022 |
| Sex |  | 0.007 | 0.729 | 0.58 | 0.916 |
| ASA Class |  | 0.329 | 1.154 | 0.866 | 1.537 |
| Minor vs Major Hepatectomy |  | < 0.001 | 1.851 | 1.442 | 2.375 |
| Type of Colorectal Resection | |  |  |  |  |
|  | Right Colectomy (ref) | 0.039 |  |  |  |
|  | Left colectomy | 0.367 | 1.133 | 0.864 | 1.485 |
|  | Proctectomy | 0.119 | 0.788 | 0.584 | 1.063 |
| Laparoscopic vs Open | | < 0.001 | 2.701 | 1.666 | 4.379 |
| Constant |  | < 0.001 | 0.01 |  |  |
